# Extending certified spectral fluorescence standards for the calibration and performance validation of fluorescence instruments to the NIR—closing the gap from 750 to 940 nm with two novel NIR dyes

**DOI:** 10.1007/s00216-024-05723-w

**Published:** 2025-02-12

**Authors:** M. Richter, A. Güttler, J. Pauli, K. Vogel, C. Homann, C. Würth, U. Resch-Genger

**Affiliations:** 1 Division Biophotonics, Federal Institute for Materials Research and Testing (BAM), Richard-Willstaetter-Str. 11, Berlin, D-12489 Germany; 2 Division eScience, Federal Institute for Materials Research and Testing (BAM), Unter den Eichen 87, Berlin, D-12205 Germany

**Keywords:** Fluorescence standard, Near infrared, Dye, Certification, Calibration, Uncertainty budget, Traceability

## Abstract

Fluorescence techniques such as fluorescence spectroscopy, microfluorometry, and fluorescence microscopy, providing spectral, intensity, polarization, and lifetime information, are amongst the most broadly utilized analytical methods in the life and materials sciences. However, the measured fluorescence data contain sample- and instrument-specific contributions, which hamper their comparability across instruments and laboratories. Comparable, instrument-independent fluorescence data require the determination of the fluorescence instrument’s wavelength-dependent spectral responsivity, also termed emission correction curve, for the same instrument settings as those used for the fluorescence measurements as a prerequisite for the subsequent correction of the measured instrument-specific data. Such a spectral correction is essential for the performance comparison of different fluorescent labels and reporters, quantitative fluorescence measurements, the determination of the fluorescence quantum yield, and the spectroscopic measure for the fluorescence efficiency of a fluorophore. Simple-to-use tools for obtaining emission correction curves are chromophore-based reference materials (RMs), referred to as fluorescence standards, with precisely known, preferably certified instrument-independent fluorescence spectra. However, for the increasingly used near-infrared (NIR) wavelength region >700 nm, at present, no spectral fluorescence standards are available. To close this gap, we developed two novel spectral fluorescence standards, BAM F007 and BAM-F009, with broad emission bands from about 580 to 940 nm in ethanolic solution. These liquid fluorescence standards currently under certification, which will be released in 2025, will expand the wavelength range of the already available certified *Calibration Kit* BAM F001b-F005b from about 300–730 to 940 nm. In this research article, we will detail the criteria utilized for dye and matrix selection and the homogeneity and stability tests accompanying dye certification as well as the calculation of the wavelength-dependent uncertainty budgets of the emission spectra BAM F007 and BAM-F009, determined with the traceably calibrated BAM reference spectrofluorometer. These fluorescence standards can provide the basis for comparable fluorescence measurements in the ultraviolet, visible, and NIR for the fluorescence community.

**Supplementary Information:**

The online version contains supplementary material available at 10.1007/s00216-024-05723-w.

## Introduction

Inherently multiparametric fluorescence techniques^1^ such as fluorescence spectroscopy, microfluorometry, and fluorescence microscopy are broadly utilized in the life and materials sciences [1–4]. Fluorescence measurements can provide spectral, intensity, lifetime, and polarization information on a fluorophore in its environment with an unmatched high sensitivity down to the single molecule level and can be performed with relatively inexpensive, broadly available, and readily miniaturized instrumentation that is often simple to use [1, 4, 5]. However, each method and device measuring fluorescence either spectrally resolved or integrally yield sample- and instrument-dependent data which cannot be directly compared with the results obtained with a different instrument [3, 4, 6]. Rendering fluorescence data comparable between different instruments and laboratories requires to consider instrument-specific signal contributions and distortions caused by the wavelength-dependent properties of the instrument’s optical and opto-electric components such as excitation light sources, monochromators, polarizers, and detectors [7, 8]. Instrument properties to be assessed by reliable calibration procedures include the wavelength dependence of the spectral responsivity of the detection channel (also referred to as emission correction) for fluorescence emission spectra and fluorescence quantum yields, and the wavelength dependence of the spectral radiant power of the instrument’s excitation channel (termed excitation correction) for fluorescence excitation spectra [7, 9]. The latter equal the absorption spectra of the emissive species. In addition, tools are needed for the regular control of instrument performance and calibration to detect, monitor, and consider changes in the spectral responsivity of fluorescence instruments caused by the aging of optical and opto-electric instrument components.

Fluorescence instruments can be calibrated with physical devices, often referred to as physical transfer standards, or with chemical reference materials (RMs), termed fluorescence standards, with precisely known fluorescence properties [7, 8]. Typical examples for the former are source-based standards such as tungsten strip lamps or less intense calibration lamps, constituting of a halogen lamp placed in an integrating sphere, utilized for the determination of the wavelength-dependent spectral responsivity of the detection channel of fluorescence instruments [10]. These source-based standards typically come with a certificate of their wavelength-dependent spectral radiance including wavelength-dependent uncertainty statements. The spectral radiant power of such calibration lamps, however, exceeds that emitted by luminescent samples by several orders of magnitude [11]. Therefore, a source-based calibration with identical instrument settings as used for measurements of luminescent samples, which is mandatory for traceable fluorescence measurements, typically requires the attenuation of the calibration lamp without introducing additional spectral effects or signal distortions due to detector nonlinearities. In addition, the accurate handling of calibration lamps can be challenging and demands a profound knowledge in optics. Alternatively, RMs such as spectral fluorescence standards with precisely known emission spectra can be employed [8, 11, 12]. Usage of such much less intense and simpler to use chromophore-based RMs, which often more closely match common samples, can elegantly circumvent such sources of uncertainty. Fluorescence standards are hence the logical choice for many users of fluorescence techniques to establish and verify the reliability of their in-house fluorescence measurements, which is essential, e.g., for industry acceptance, accreditation, and regulatory approval [13, 14]. Examples for commercial spectral fluorescence standards for the calibration, characterization, and qualification of spectrofluorometers are organic or inorganic emitters in different matrices such as a certified set of spectrally matched dye solutions [15, 16], certified cuvette-shaped glasses doped with luminescent metal ions [17–20], provided by national metrology institutes, i.e., BAM and the National Institute of Standards and Technology (NIST), or dye-stained polymer blocks from an industrial manufacturer (Starna Scientific Ltd, Fluorescence Reference Materials, 58F). These fluorescence standards currently cover the wavelength range of about 300 to 800 nm.

In the last two decades, the utilized spectral window of fluorescence measurements continuously increased and shifted to longer wavelengths, triggered and flanked by the development of an increasing number of luminophores emitting above 650 nm in the near infrared (NIR) and affordable fluorescence measuring devices with an improved sensitivity in the NIR [21–26]. This window has been meanwhile expanded to about 1000 nm as the strongly reduced autofluorescence and scattering in the NIR compared to the ultraviolet (UV) or visible (vis) wavelength region can enable improved signal-to-background signals and hence a higher analyte sensitivity, particularly for measurements in complex biological matrices. This calls for simple calibration strategies and easy-to-use NIR fluorescence standards to establish the traceability of fluorescence measurements in the NIR to a radiometric or photonic scale such as the spectral radiance or spectral photon radiance scale.

In this research article, focusing on chromophore-based RMs, the development and certification of two novel fluorescence standards with emission bands covering the wavelength range of 580 to 940 nm are described including homogeneity and stability tests and the determination of the respective wavelength-dependent uncertainty budgets. In this context, also general requirements on fluorescence standards and design and quality criteria are provided including the choice of fluorophore-matrix combinations with special emphasis on spectral fluorescence standards.

## Materials and methods

### Materials

The starting materials of CRMs BAM-F07 and BAM-F009 were purchased from Exciton, Lockbourne, USA. All dyes were employed without further purification. Absolute ethanol Chromosolv™ for HPLC (purity ≥99.8%) from Honeywell/Riedel de Haen (Art. Nr. 34852-1L) was used as solvent.

### Instrumentation

#### Absorption spectrometer CARY 5000

Absorption measurements of the dye solutions were done with the spectrophotometer CARY 5000 (Varian Inc.) at T = 25 °C. Instrument validation is performed with a set of calibrated secondary standards consisting of one holmium oxide glass filter for wavelength accuracy and three neutral glass filters with different optical densities for intensity calibration. Type 666S000 was manufactured by Hellma GmbH & Co. KG.

#### Spectrofluorometer SLM (Aminco-Bowman Inc)

For the determination of the homogeneity of the bottled fluorescence standards, the fluorescence spectra of dye solutions from selected bottles were determined using with a step size of 1 nm and an integration time of 1 s/point. The polarizers in the excitation and emission channel were set to 0° and to 54.7° in the emission channel, respectively. All measurements were done at T = 25 °C.

#### Spectrofluorometer FLS920 (Edinburgh Instruments)

The measurements of the bottled samples of BAM-F007 and F009 were carried out with Glan-Thompson polarizers in the excitation and emission channel set to 0° and 54.7° using 1-nm data intervals and photon counting detection. For detection of the fluorescence emission spectra, the PMT detector R2658P and gratings of the emission monochromator with 1200 lines/blaze 750 nm were used. A reference detector (Si-diode) was employed for monitoring the fluctuation of the continuous Xe-light source. The chosen slit of the excitation monochromator for the certification measurements was selected to guarantee that the resulting photon counting rates at the emission detector did not exceed 10 [6] counts/s. All fluorescence measurements were carried out using a temperature-controlled sample holder (set to T = 25 °C).

#### Calibration tools

The traceable wavelength-dependent relative spectral responsivity of the spectrofluorometer FLS 920 was determined by illuminating a Lambertian reflecting diffuser (white standards; calibrated spectral radiance factor) at an incident angle of 45° with a calibrated spectral radiance standard, referred to here as SRS. The Lambertian diffuse reflecting PTFE reflectance standard (45° bevelled cuboid, Berghof Fluoroplastic Technology) has a known 45°/0° spectral radiance factor calibrated by the National Physical Laboratory (NPL), UK (NPL certificate 2020010110). The SRS is an integrating sphere radiator BN-9701 (10-W quartz halogen bulb, Gigahertz-Optik) calibrated by the Physikalisch-Technische Bundesanstalt (PTB) (PTB certificate 73124 PTB 22).

### Methods: Dye configuration, homogeneity and stability studies, and uncertainty calculation

#### Configuration of the NIR spectral standards BAM-F007 and BAM-F009

The configuration of BAM-F007 and BAM-F009 included the preparation, i.e., bottling of 480 samples of each dye. Therefore, a defined quantity of each dye was dissolved in 500 mL of absolute ethanol in an ultrasonic bath. Then, 480 white glass bottles with a volume of 15 mL were each filled with 1 mL of the respective dye solution and the solvent was evaporated in vacuo at 30°C. The bottles containing the dried dyes were then filled with an argon cushion and stored at 4°C; additionally 60 bottles are stored at −20°C.

#### Preparation of the dye solutions for the homogeneity and stability studies

Solutions of each candidate RM with an absorbance (A) of 0.04 at the recommended excitation wavelength were used for the homogeneity and stability studies. The preparation of these solutions was done in three steps. Ten milliliters of ethanol was added to each selected bottle containing the solid dye yielding a dye stock solution. Each bottle was placed in an ultrasonic bath for 5 min to speed up dye dissolution. This stock solution was then twice diluted. Thereby, 500 µL of the dye stock solutions was diluted in 2500 µL ethanol (*A* = 0.4). In the second dilution step, the absorbance of the dye solutions was adjusted to 0.04 or 0.12 at the recommended excitation wavelengths of 600 nm for BAM-F007 and 590 nm for BAM-009.

#### Homogeneity testing of the spectral shape

To assess the homogeneity of the spectral shape of the fluorescence emission spectra of dyes BAM-F007 and BAM-F009, 16 bottles of each fluorophore stored at 4 °C were evaluated, in agreement with ISO 33405:2024 [27] wherein a homogeneity assessment is required for the minimum number of samples (${N}_{\text{min}}=\text{max}\left(10, \sqrt[3]{480}\right)=10)$ of each RM batch consisting of 480 bottles. The freshly prepared dye solutions and the solvent ethanol used as a blank were spectroscopically assessed utilizing identical instrument settings of the calibrated spectrofluorometer. For the assessment of the homogeneity of the spectral shape of the spectrally corrected emission spectra, the fluorescence spectra were corrected for signal contributions from the solvent (subtraction of the solvent signal recorded under identical measurement conditions, termed blank correction) and zero-offsets if needed, and then normalized at the respective emission maximum. In the case of BAM-F009, the recorded fluorescence spectra were smoothed using the Savitzky-Golay smoothing filter prior to normalization to reduce the influence of the low signal to noise ratio of the measured data.

For the evaluation of the between-unit standard deviation, ${s}_{\text{bb}}$ calculated from the within- and between-unit variances (*MS*) according to ISO 33405:2024, was used to control for the significance of these variations using the *p*-value of the performed ANOVA of each data set.

1 $${s}_{\text{bb}}= \sqrt{\frac{{MS}_{\text{between}}- {MS}_{\text{within}}}{n}}$$

where:

$${MS}_{\text{between}}$$
Mean of squared deviations between the intensities of the solutions of the different bottles at a selected wavelength (from one-way ANOVA)
$${MS}_{\text{within}}$$
Mean of squared deviations between the intensities of different solutions of one vial at a selected wavelength (from one-way ANOVA)
$$n$$ 
Number of replicate measurements per bottle, here *n* = 2

The dye-specific inhomogeneity contribution *u*_r_bb_ for BAM-F007 and BAM-F009 was calculated according to ISO 33405:2024 [27], using Equations 2 and 4:

2 $${u}_{\text{bb}}=\text{max}({s}_{\text{bb}}, {s}_{\text{bb}\_\text{min}})$$

3 $${\text{with}: s}_{\text{bb}\_\text{min}}= \sqrt{\frac{{MS}_{\text{within}}}{n}} \sqrt[4]{\frac{2}{N\left(n-1\right)}}$$

4 $${u}_{\text{r}\_\text{bb}}=\frac{{u}_{\text{bb}}}{\text{Total mean intensity }(n, N)}$$

where:

$${s}_{\text{bb}}$$
Dye-specific inhomogeneity contribution (Eq. 1) = between-unit standard deviation
$${MS}_{\text{within}}$$
Mean of squared deviations between the intensities of different solutions of one vial at a selected wavelength (from one-way ANOVA)
$$n$$ 
Number of replicate measurements per vial, here *n* = 2
*N*: Number of bottles selected for homogeneity study, here *N* = 13 for BAM-F007 and *N* = 16 for BAM-F009

#### Long- and short-time stability

The selected criterion for assessing the stability of the spectral shape of the solutions of the bottles containing BAM-F007 and BAM-F009 is the mean standard deviated spectrum ${dSp}_{{\text{t}}_{i}}^{\text{T}}(\lambda )$, defined as multiple of the standard deviation of the mean of the reference values, related to the Z-score. It is calculated using Eq. 5 for all wavelengths assessed.

5 $${dSp}_{{\text{t}}_{i}}^{\text{T}}(\lambda ) = \frac{\overline{{I }_{{\text{t}}_{i}}^{\text{T}}(\lambda )}- \overline{{I }_{{\text{t}}_{i}}^{-20^\circ \text{C}}(\lambda )}}{{SD}_{{\text{t}}_{i}}^{-20^\circ \text{C}}(\lambda )}$$

where:

$$\overline{{I }_{{\text{t}}_{i}}^{\text{T}}(\lambda )}$$
Standardized mean spectrum of the solution of samples stored at a defined temperature (T = 4 °C) over a defined period of time *t*_i_
$$\overline{{I }_{{\text{t}}_{i}}^{-20^\circ \text{C}}(\lambda )}$$
Standardized mean reference spectrum of the solution of the standards stored at −20 °C for the same period of time *t*_i_
$${SD}_{{\text{t}}_{i}}^{-20^\circ \text{C}}(\lambda )$$ 
Standard deviation of the standardized reference spectra

### Design criteria for fluorescence standards for the UV/vis/NIR

A chromophore-based RM should have the following properties [12]: (i) simple to use, excitable with common light sources, and measurable with typical instrument settings; (ii) display a spectral shape of its fluorescence emission or excitation spectrum suitable for its scope; (iii) exhibit application-relevant fluorescence properties that do not depend on excitation wavelength, i.e., a constant fluorescence quantum yield independent of excitation wavelength originating from a single absorption band; (iv) show a sufficient stability under application-relevant conditions, i.e., typical temperatures and excitation power densities, and should not form an absorbing or emitting photoproduct; and (v) have a small emission anisotropy. The latter is a measure for the response of the fluorescence properties of a fluorophore to excitation with (partly) polarized light and relevant for measurements with fluorescence instruments lacking polarizers. Also, (vi) the temperature dependence of the standard’s fluorescence properties in the typical room temperature range should be known and should be preferably small. As additional information and documentation, (vii) the standard’s storage conditions should be provided and its shelf life as well as (viii) a standard operating procedure (SOP) for its handling and usage.

For all applications, which require traceable fluorescence measurements and hence, a traceable instrument calibration or performance validation, e.g., for laboratories accredited according to ISO/IEC 17025, (ix) a traceability statement is required. Metrological traceability is defined as a property of a measuring result whereby the result can be related to a reference through a documented unbroken chain of calibrations, each contributing to the measurement uncertainty [28]. Typically, traceability to SI units (Système international d’unités = International System of Units) is established during the certification procedure of the RM, commonly via the calibration of the instruments used for determination of the certified property, i.e., the corrected fluorescence emission spectra of spectral fluorescence standards. Hence, traceable fluorescence measurements require high-quality RMs and the measurement of the standard’s application-relevant fluorescence properties with a traceably calibrated spectrofluorometer, the calibration of the which should be well documented [29], and the provision of uncertainty statements [12, 30, 31] that are wavelength-dependent for fluorescence spectra. In this context, it should be differentiated between RMs and certified reference materials (CRMs). A RM is sufficiently homogeneous and stable with respect to one or more specified properties, which are suited for its intended use, while a CRM is a material characterized by a metrologically valid procedure for one or more specified properties, accompanied by a certificate that provides the value of the specified property, its associated uncertainty, and a statement of metrological traceability [32]. Commercial producers and suppliers of RMs may use different, e.g., less time-consuming and expensive procedures for material characterization, that can be less metrologically rigorous, hampering traceability, but sometimes nevertheless classify their RMs as “certified,” despite not having followed the ISO requirements.

### Criteria for fluorophore and matrix selection

The design of a broadly applicable fluorescence standard requires the choice of a suitable chromophore and a suitable matrix as the absorption and fluorescence properties of most fluorophores depend on their environment, especially on properties such as polarity, proticity, and viscosity, as well as on temperature.

#### Chromophore choice

Spectroscopic properties relevant for chromophore selection include (i) the spectral position of the absorption and emission band and the spectral overlap between absorption and emission, termed Stokes shift and (ii) the spectral shape and spectral width of the emission spectrum, i.e., whether the fluorescence band has a vibronic fine structure, which is typical for planar organic dyes with a fluorescence from a locally excited (LE) state, is line shaped as is the case for many lanthanide ions, or broad as displayed by charge transfer (CT)–operated organic dyes in polar media [1, 5]. Also, (iii) the chromophore’s fluorescence quantum yield including a possible dependence on excitation wavelength, and (iv) the fluorescence lifetime should be considered. The latter can be relevant for the occurrence of polarization effects and the magnitude of the emission anisotropy. In addition, it can affect fluorescence measurements with instruments using pulsed light sources, especially for a lifetime in the ms- to ms range as revealed, e.g., by emitters such as lanthanides [1]. (v) Chromophores, that reveal a strong temperature dependence in the typical room temperature range of about 20 to 30°C, requiring precise temperature control, or a strong excitation wavelength dependence of their fluorescence properties, restricting their usage to defined excitation wavelengths, are not an optimum choice.

#### Matrix choice

(vi) Matrix viscosity: Organic dyes with nanosecond fluorescence lifetimes always show an anisotropic emission in solid matrices such as polymers, where rotations are hindered, rendering the use of polarizers mandatory to avoid additional uncertainty contributions [1], while other solid materials such as glasses doped with luminescent lanthanide or transition metal ions typically still reveal a very small emission anisotropy [17–20, 33]. In addition, (vii) a possible influence of environment humidity (water uptake) on matrix properties or stability, requiring, e.g., storage in a desiccator for certain glasses [17–20], should be considered as well as (viii) mechanical stability or other features, possibly affecting the standard’s application-relevant fluorescence properties, storage conditions, or short-term (transport) or long-term stability.

#### Liquid vs. solid standards

A liquid fluorescence standard often requires a preparation step such as the dissolution of a solid chromophore and the dye solution has a limited shelf life. Advantages are a homogeneous chromophore distribution, flexibility with respect to emitter concentration, and ease of format adaptation, enabling the usage of liquid standards for calibrating and characterizing different types of fluorescence instruments such as fluorescence spectrometers, microplate readers, and fluorescence microscopes [34]. In addition, dye solutions closely resemble typically measured liquid samples. A solid fluorescence standard such as a chromophore-stained polymer block or slide or fluorophore-doped glass can be used without a preparation step and is designed for longer term usage. However, its use is limited to a fixed measurement geometry and hence a specific type of fluorescence instrument, and the fluorophore concentration cannot be modified. In addition, the homogeneity of the emitter distribution must be controlled as this can introduce spatial variations in fluorescence. Solid standards can also be prone to polarization effects and local photobleaching.

**Fig. 1:**
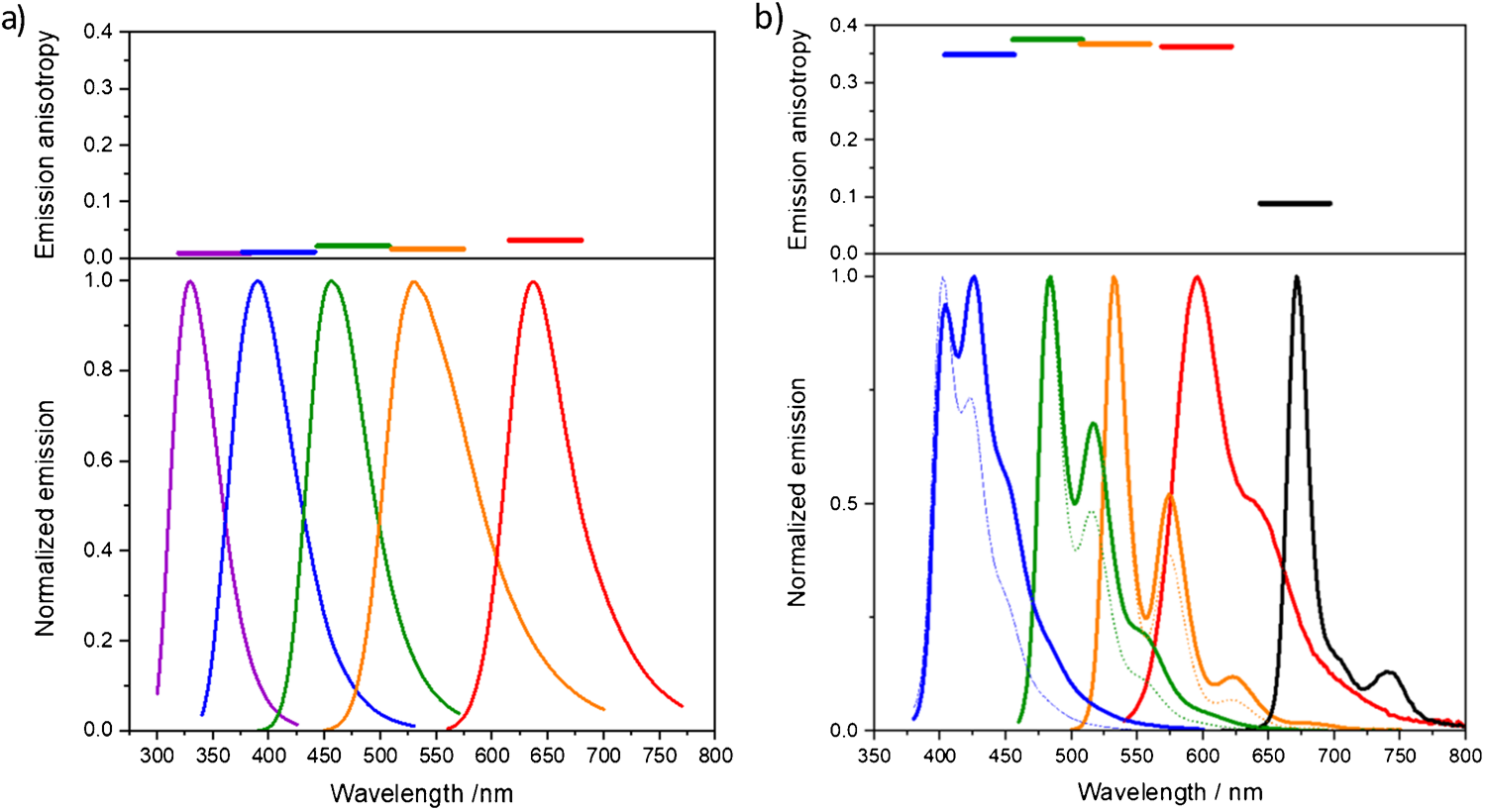
**a** Normalized corrected emission spectra (bottom) and emission anisotropy (top) of the certified spectral fluorescence standards BAM-F001-F005 in ethanol and **b** normalized corrected emission spectra (bottom) and emission anisotropy (top) of the Starna polymer blocks measured with the calibrated BAM fluorometer FLS920 with the excitation polarizer set to 0° and the emission polarizer to 54.7° (solid lines) and without polarizers (dotted lines), respectively

Chromophores with very narrow, line-shaped emission bands at precisely known wavelength positions or mixtures of such chromophores are best suited candidates for simple wavelength standards and instrument performance validation (IPV) standards [12, 33]. The design of spectral fluorescence standards for the determination of the spectral responsivity of fluorescence instruments, however, requires emitter-matrix combinations that provide broad and unstructured fluorescence emission spectra as thereby, a dependence on the spectral bandpass of the fluorescence instrument to be calibrated is minimized [11, 12]. To cover a very broad spectral window such as the UV/vis/NIR, fluorophore combinations or standard sets are needed [11, 15, 35]. Such sets should be designed to reveal a certain spectral overlap to ease the generation of a spectral correction curve for UV/vis/NIR [11, 31, 35]. Figure 1 displays the fluorescence emission spectra of representatively chosen, commercial liquid and solid fluorescence standards. The left panel of this figure shows the certified corrected emission spectra of five dyes, dissolved in ethanol, with spectrally matched, broad, and certified corrected emission spectra [11, 15], obtained with a traceably calibrated fluorometer with a complete uncertainty budget, which were assessed in a first international interlaboratory comparison (ILC) of four metrology institutes [31]. The emission anisotropies of these dye solutions are below 0.05 (Fig. 1, left, top), rendering the usage of polarizers not mandatory. In the right panel of Fig. 1, the emission spectra of five commercial cuvette-shaped dye-stained polymer blocks are shown. All five fluorophores display considerably structured emission spectra and a very high emission anisotropy (Fig. 1, right, top). For these standards, uncertainty statements are missing, hampering traceability. The high emission anisotropy may cause problems in devices working without polarizers (see Fig. 1).

## Results and discussion

In this research article, focusing on spectral fluorescence standards and especially on the increasingly important NIR wavelength region, the development, characterization, and certification of the novel fluorescence standards BAM-F007 and BAM-F009 with emission bands covering the wavelength range of 580 to 940 nm is presented. This also includes the determination of the wavelength-dependent uncertainty budgets of the certified corrected fluorescence emission spectra. The criteria utilized for the development of broadly applicable chromophore-based RMs, which enable the traceable determination of the spectral responsivity of fluorescence measuring devices, have been provided in the previous section.

### Design and production of certified spectral NIR fluorescence standards

The production of a RM or CRM as described in the ISO 17034 (2017) [36], ISO Guide 31:2015 [37], and ISO 33405:2024 [27] involves the following steps: (a) definition of the RM, i.e., the measurand and matrix, the properties to be characterized, and the method of choice, the desired target range and uncertainty for the property value of each method, the intended use or scope of the material, and for CRMs, the target uncertainty. Then, procedures must be developed for (b) material sourcing and (c) RM preparation, followed by (d) the selection of measurement procedures suitable for characterization, homogeneity, and stability studies. For CRMs, also (e) establishing metrological traceability for each measured property is required, typically by appropriate instrument calibration. Subsequently, (f) the homogeneity of the packaged material, (g) its stability, and if required, also (h) the commutability must be assessed. Finally, (i) the RM is accordingly characterized and (j) the results from homogeneity studies and stability studies, characterization measurements, and instrument calibrations are combined and (k) utilized for the calculation of the uncertainty budget of the targeted RM property according to the Guide for the Measurement of Uncertainties (GUM) [38], especially for the certified values. This information is provided as a certificate or product information sheet including (l) specified storage and transportation conditions. Commonly, also (m) a post-production monitoring of RM stability is performed utilizing defined storage conditions and measuring scope-relevant properties.

### Dye selection—BAM-F007 and BAM-F009

**Fig. 2:**
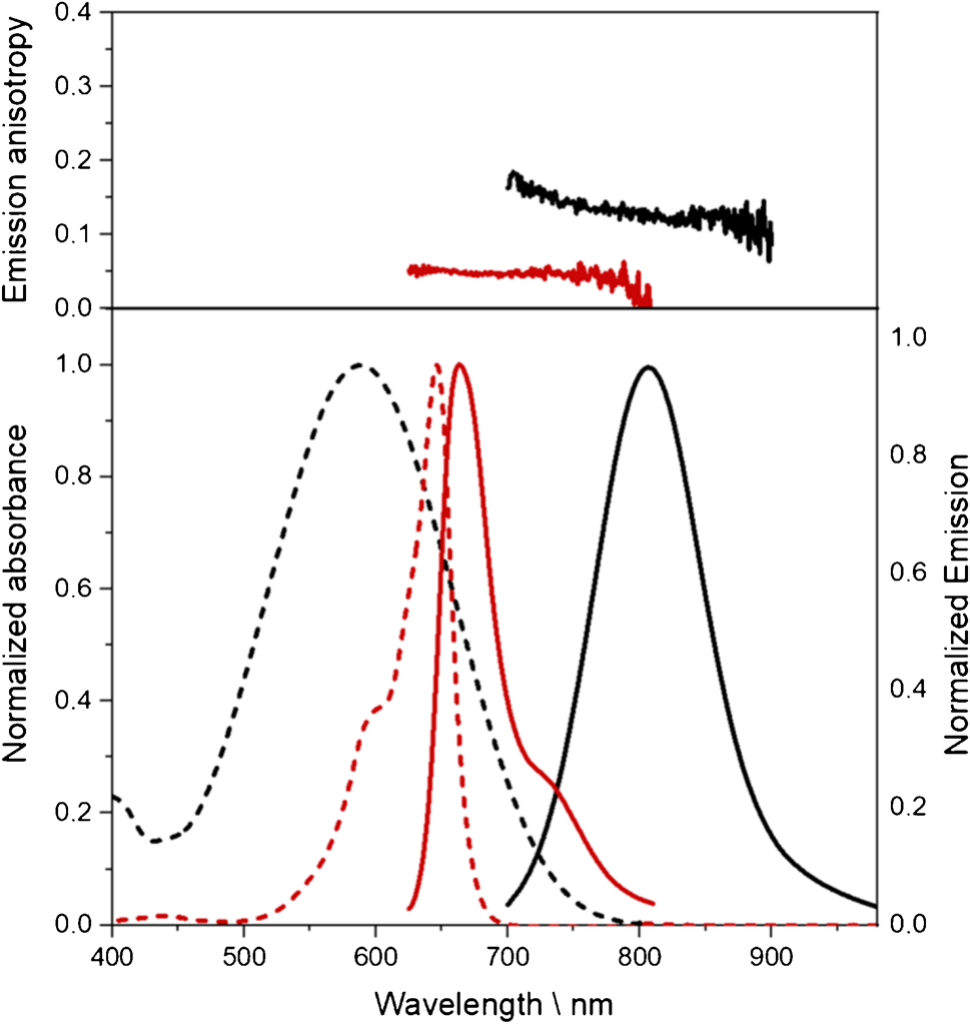
Top: emission anisotropy and bottom: normalized absorbance (dashed line) and emission (solid lines) spectra of BAM-F007 (dark red) and BAM F009 (black)

To extend the wavelength region accessible for the calibration and performance validation of fluorescence instruments with fluorescence standards to the NIR region, we developed the two NIR emissive fluorescence standards, BAM-F007 and BAM-F009, thereby considering the previously presented design criteria for broadly applicable fluorescence standards. The normalized absorption and corrected fluorescence emission spectra of these dyes in ethanolic solution are shown in Fig. 2. These fluorophore-matrix systems were chosen according to the previously detailed design criteria, thereby considering the typical fluorescence properties of organic NIR fluorophores. For the NIR wavelength region, however, some compromises and adaptations of certain selection criteria were necessary. For example, CT-operated NIR-emissive dyes with broad emission bands and an at least moderate fluorescence quantum yield > 0.1 are very rare and the vast majority of NIR fluorophores reveals slightly structured emission bands. In addition, the larger chromophore p-systems of NIR fluorophores together with the, compared to dyes with emission in the UV and vis, reduced fluorescence lifetime, favor a slightly increased emission anisotropy of solutions of such dyes even in non-viscous solvents such as ethanol (Fig. 2, top). [1]

Other criteria for dye selection included (i) the solubility in ethanol, as a non-toxic solvent broadly available in a high quality, (ii) overlapping emission bands of both dyes, and (iii) for the shorter wavelength dye BAM-F007, an emission band slightly overlapping with that of the certified spectral fluorescence standard BAM-F005, the longest wavelength dye of the certified spectral *Calibration Kit* of BAM. This is a prerequisite to extend the wavelength region covered by the spectral *Calibration Kit* from currently 300–750 up to 940 nm, as a spectrally matched UV/vis/NIR dye set. In addition, (iv) the chosen dyes should show excitation wavelength independent emission spectra and (v) be excitable also with lasers or laser diodes typically employed for fluorescence microscopy and imaging studies, and (vi) available in a high purity > 99%. A lower dye purity could complicate homogeneity studies of the bottled standard samples during the certification process and could have made additional purification steps necessary.

After having spectroscopically assessed several NIR fluorophore candidates, we opted for BAM-F007 and BAM-F009 as optimum compromises. The suitability of the chosen spectral standard candidates was then evaluated by spectroscopic studies of these fluorophores, dissolved in ethanol. Thereby, also photostability studies with dye solutions containing dye concentrations, which are typically used for fluorescence measurements, were performed, employing an excitation power comparable to the excitation intensities provided by commercial spectrofluorometers and integrating sphere setups in the order of 1.0E-05 up to 7.0E-05 W at the excitation wavelengths recommend for dye usage. Under these conditions, ethanolic solutions of BAM-F007 and BAM-F009 revealed no illumination-induced changes of their emission spectra after 17 h of continuous illumination.

### Certification of BAM-F007 and BAM-F009

#### Homogeneity testing of the spectral shape

**Fig. 3:**
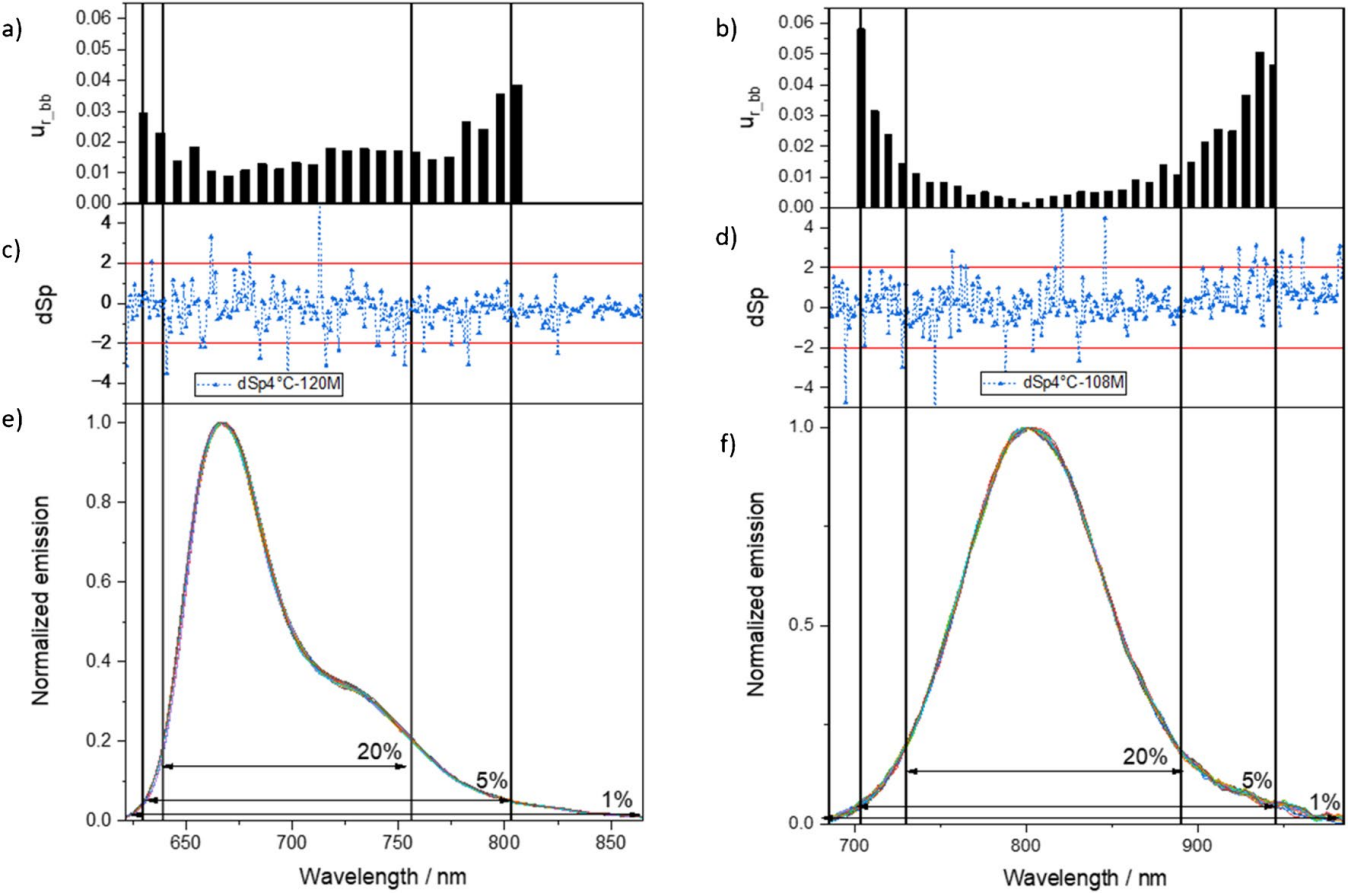
Dye-specific relative uncertainty due to the inhomogeneity (*u*_r_bb_*(λ*)) of the fluorescence spectra of **a** BAM-F007 (*k* = 1, i.e., confidence interval of 68%), relevant wavelength range specified for the use of the respective dye as spectral fluorescence standard: 639–756 nm with *u*_r_bb_ < 0.02 (< 2%) and **b** BAM-F009 (*k* = 1, i.e., confidence interval of 68%), relevant wavelength range specified for use of this dye as spectral fluorescence standard: 730–890 nm with *u*_r_bb_ < 0.02 (< 2%). Mean standard deviated spectrum ${dSp}_{{\text{t}}_{120\text{M}}}^{\text{T}}\left(\lambda \right)$ of the emission spectra of **c** BAM-F007 after storage time 120 months at time of certification and **d** BAM-F009 after storage time 108 months at time of certification. Emission spectra of **e** BAM-F007 and **f** BAM-F009 with corresponding intensity percentages of 1%, 5%, and 20%

For the evaluation of the homogeneity of the spectral shape of the RM materials, the one-way ANOVA was performed at selected wavelengths sorted by sample bottles and replicate measurements per bottle. The selected wavelengths were evenly distributed over the spectral range of the measured fluorescence spectra, at every 8 nm starting and ending with the wavelength, which had an intensity above 5% of the maximum intensity (see Fig. 3). The homogeneity of each data set (intensity values per bottle and repetition) was then tested utilizing a significance level of *α* = 0.05.

The contribution ${s}_{\text{bb}}$ (see the “Materials and methods” section) has only to be considered if the between unit variation ${MS}_{\text{between}}$ is larger than (or minimally equal to) the within unit variation ${MS}_{\text{within}}$. In the latter, case ${s}_{\text{bb}}$ is set to 0. ${s}_{\text{bb}}$ was calculated for the emission intensity data set at each selected wavelength according to Eq. 1. The significance of the inhomogeneity if ${s}_{\text{bb}}$ > 0 was tested with the *p*-value of the ANOVA with *p* > 0.05 confirming “non-significant inhomogeneities.” The final evaluation of the homogeneity of the spectral shape was done for fluorescence intensities exceeding 20% of the maximum intensity (see Fig. 3), thereby only considering the application-relevant wavelength range of the CRM to be used for instrument calibration and characterization. Ninety-four percent of the tested fluorescence intensity intervals showed no inhomogeneities for BAM-F007 and 95% for BAM-F009. This indicates that the spectral shape of the emission spectra of BAM-F007 and BAM-F009 is homogeneous within the entire relevant wavelength range. The relative dye-specific inhomogeneity contribution *u*_r_bb_ (see also the “Materials and methods” section) to the uncertainty budget was considered in the 5% intensity interval (see Fig. 3a and b).

### Long- and short-time stability

The spectral shape of the emission spectra of the different bottles of BAM-F007 and BAM-F009 is regarded as unchanged if the ${dSp}_{{\text{t}}_{i}}^{\text{T}}(\lambda )$ (see “Materials and methods”) in a defined wavelength range reveals values between −2 and 2, with $\left|2\right|$ being the tolerance limit of the measured intensity value at the considered wavelength, within the accepted confidence interval of 95% of the corresponding intensity value of the reference spectrum. If 5 or more consecutive ${dSp}_{{\text{t}}_{i}}^{\text{T}}(\lambda )$ values in the defined wavelength range exceed this value or are equal to $\left|2\right|$, the spectral shape is considered as changed and an uncertainty contribution due to this spectral change or instability must be considered.

For the calculation of ${dSp}_{{\text{t}}_{i}}^{\text{T}}$, the measured spectra must be standardized regarding their intensity values to exclude deviations originating from sample preparation steps, inhomogeneities, or instrument effects. Therefore, the standardization factors *Fi* were calculated using the following steps:

1. Calculation of the mean reference spectrum: $\overline{{I }_{{\text{t}}_{i}}^{-20^\circ \text{C}}(\lambda )}$
2. Smoothing of the mean reference spectrum and all measured fluorescence spectra for all storage temperatures and repeated measurements using the Savitzky-Golay smoothing filter
3. Determination of the max value of the smoothed mean reference spectrum and all individual fluorescence spectra.
4. Calculation of the individual *Fi*-factors by dividing the individual emission spectrum by the maximum value of the mean reference spectrum of all measured fluorescence spectra.
5. Multiplication of each measured spectra derived for all storage temperatures and repeated measurements with their *Fi*-factor (non-smoothed spectra).

The evaluation of the stability of the dried solid dyes was done for all ${dSp}_{{\text{t}}_{i}}^{\text{T}}(\lambda )$ values within the application-relevant wavelength range with intensity values exceeding 20% of the maximum intensity *I*_20%_(*λ*_em_). The results of the stability tests under defined storage conditions, here 4°C, are shown in Fig. 3c and d in the lower panels. As follows from these data, the emission spectra of BAM-F007 and BAM-F009 show no significant changes. Hence, the long-term stability of these fluorescence standards is given when stored at 4°C. Comparable results were obtained for the short-time stability studies of the prepared stock solutions, the stability of which was studied over a period of 3 months. The stability studies do not contribute to the overall uncertainty and were not considered for the calculation of the uncertainty budgets of the emission spectra of BAM-F007 and BAM-F009. Post-certification stability monitoring is currently done in regular intervals to ensure the stability of both CRMs.

### Statistical evaluation and calculation of the certified values

**Fig. 4:**
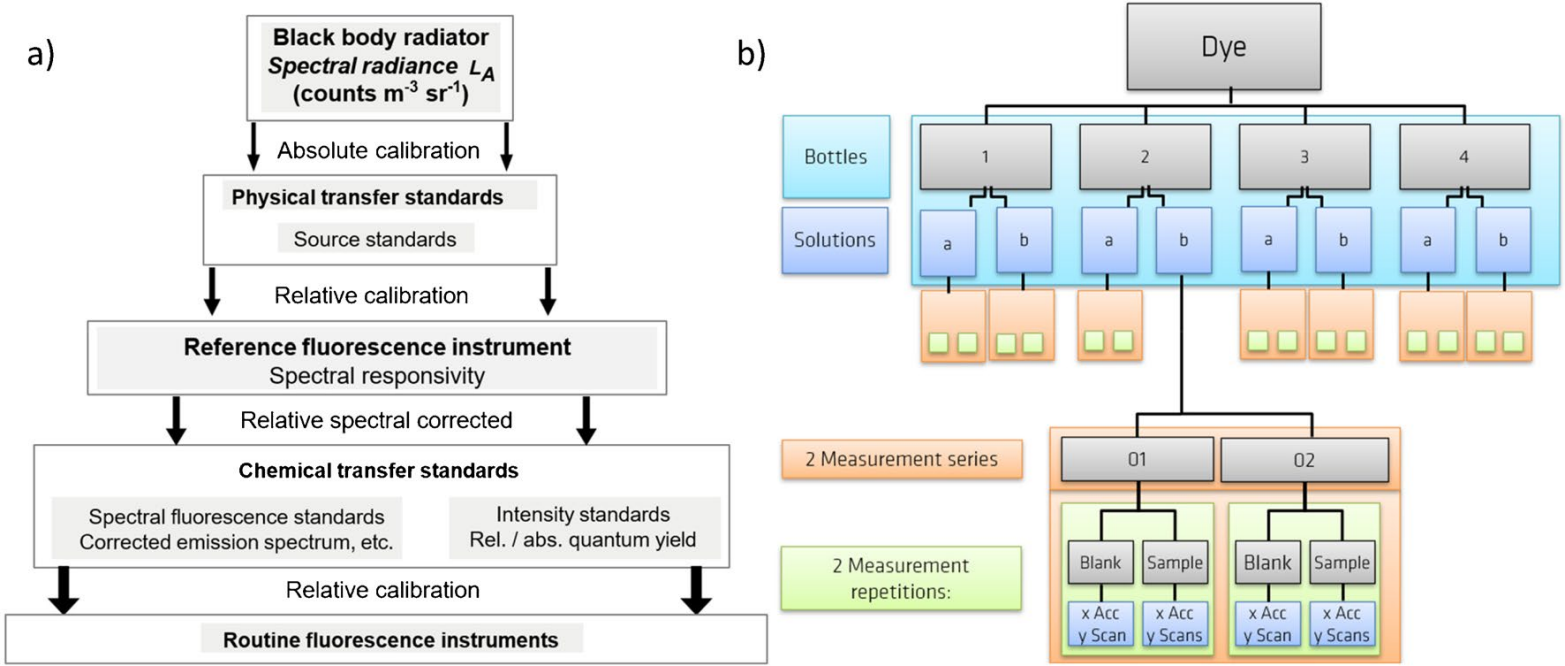
**a** Traceability chain of the spectral CRMs. **b** Scheme of the certification measurement procedure

The determination of the subsequently certified normalized corrected fluorescence emission spectra *I*_C_(*λ*_em_) of BAM-F007 and BAM-F009 was done with the calibrated spectrofluorometer FLS920 (see the “Materials and methods” section). The traceable calibration of the emission channel of this spectrofluorometer includes the determination of the wavelength accuracy, the linear range of the instrument’s detection system, and the wavelength-dependent spectral characteristics of the instrument’s detection channel (see Fig. 4a) relevant for or contributing to the instrument’s emission correction curve. This emission correction curve equals the relative spectral responsivity *s*(*λ*_em_) of the instrument’s detection channel as is described in the IUPAC Technical Report and the ASTM recommendations on the characterization and qualification of fluorescence Instruments*.* [7] The spectral characteristics were determined using calibrated physical transfer standards, i.e., a spectral radiance transfer standard (SRS) and a white standard (FRS1), as detailed in the “Materials and methods” section, thereby traceability to the spectral radiance scale and the respective primary standard, the black body radiator (see Fig. 3a) was established. To consider the photonic nature of the emitted light, the spectral radiance was transferred into spectral photon radiance scale (see Eq. 6). Thereby, accordingly corrected fluorescence emission spectra can be also directly utilized for the determination of fluorescence quantum yields [39]. Constants like the speed of light and Plank’s constant are neglected because of the relative character of the calibration (see Fig. 4a). For these calibrations, performed under identical instrument settings as the subsequent measurements of the emission spectra of BAM-F007 and BAM-F009, the calibrated light source SRS was diffusely reflected into the emission detection channel of the instrument via the white standard FRS1 (calibrated spectral radiance factor) placed at the sample position and illuminated with SRS at an angle of 45°. The spectral radiance factor of FRS1 was calibrated for this illumination geometry. This procedure enabled the attenuation of the source standard without introducing additional spectral contributions or loosing traceability [31]. The subsequently calculated emission correction curve *s*(*λ*_em_) equals the quotient of the measured uncorrected emission spectrum of SRS, corrected for the dark current of the detector, and possible inaccuracies of the wavelength scale *I*_U_LC_(*λ*_em_). The corrected emission spectrum was then divided by the certified wavelength-dependent spectral radiance factor of FRS1 *I*_FRS1_(*λ*_em_) given in the calibration certificate, the certified emission spectrum of the SRS *I*_SRS_(*λ*_em_) given in the calibration certificate in spectral radiance (SR) [W m^−3^ sr^−1^], and the transmission of the cut-off filter determined with a calibrated photometer and the wavelength considering the photonic nature of the emitted light (see Eq. 6).

6 $${s}^{{L}_{q}}\left({\uplambda }_{\text{em}}\right)=\frac{{I}_{\text{U}\_\text{LC}}{(\uplambda }_{\text{em}})}{{I}_{\text{FRS}1}{(\uplambda }_{\text{em}}) \cdot {I}_{\text{SRS}}{(\uplambda }_{\text{em}}) \cdot {T}_{\text{Filter}}{(\uplambda }_{\text{em}})\cdot \lambda }$$

The measurements performed for the certification of the corrected emission spectra of BAM-F007 and BAM-F009 are highlighted in Fig. 4b. Possible sources of uncertainty originating from sample preparation (blue area), sample handling (orange area), and instrument variations (green area) were considered. The sample preparation was always performed directly before a fluorescence measurement series. For the certification measurements, at least *N* = 4 bottles of BAM-F007 and BAM-F009 containing the solid dyes stored at 4 °C were utilized, thereby dissolving the solid dye in 10 mL ethanol and preparing two dye solutions per bottle as detailed in the “Materials and methods” section.

The mean blank spectrum was calculated from repeated measurements of the solvent ethanol under identical conditions, i.e., with the identical instrument settings used for the spectral standards (see Fig. 4b, green). The mean fluorescence emission spectrum of a sample was calculated from all repetitively performed fluorescence measurements. The mean blank spectrum was then subtracted from each mean fluorescence spectrum of BAM-F007 or BAM-F009, yielding blank-corrected or blank-subtracted emission spectra. All blank-subtracted emission spectra per repetition and series (see Fig. 4b, green and orange) were then combined to mean blank-subtracted emission spectra per bottle (index bc). These spectra were then corrected for possible wavelength inaccuracies (see SI, index lc) and for the wavelength-dependent spectral responsivity of the detection channel of the spectrofluorometer FLS920 according to Eq. 7.

7 $${I}_{\text{sc}}\left({\lambda }_{\text{em}}\right)= {I}_{\text{u},\text{bc},\text{lc}}\left(\lambda \right) / {s}^{{L}_{q}}\left({\lambda }_{\text{em}}\right)$$

The signal-to-noise ratio of the resulting corrected emission spectra ${I}_{\text{sc}}\left({\lambda }_{\text{em}}\right)$ was improved by a Savitzky-Golay smoothing filter. The smoothed fluorescence spectra were then normalized. All averaged emission spectra obtained for one dye bottle were combined to the final normalized corrected fluorescence emission spectra *I*_C_(*λ*_em_) (see Figs. 6 and 7) of BAM-F007 and BAM-F009.

### Calculation of uncertainty budget of certified property—considering RM configuration, homogeneity and stability tests, and instrument calibration

Finally, the combined uncertainty budget was determined for the emission spectra of both spectral fluorescence standards. This wavelength-dependent uncertainty budget includes dye-specific contributions and contributions originating from the uncertainty of the spectral correction ${u}_{{s}^{\text{SRS}}}$ (see Eq. 6). All uncertainty contributions considered are summarized in Table 1.

**Table 1:** Summary of the uncertainty contributions considered for the calculation of the wavelength-dependent uncertainty budgets of the certified corrected emission spectra of BAM-F007 and BAM-F009

Uncertainty contribution	Origin of the respective uncertainty contribution
*u*_SRS-cert_	Relative uncertainty of the certified spectral radiance standard (SRS) according to its certificate
*u*_Det-Lin_	Relative uncertainty originating from nonlinearities of the detection system of spectrofluorometer FLS920 operated in the photon counting mode
*u*_FRS1_	Relative uncertainty of the certified white standard used during calibration according to its certificate
*u*_Filter_	Relative standard deviation of the mean of *n* measurements of the transmission spectrum of the cut-off filter placed in the detection channel to suppress second-order effects
*u*_sdrel,c_	Relative standard deviation of the mean of *n* measurements of the emission spectrum of the spectral radiance standard
*u*_LC,c_	Relative uncertainty of the used wavelength correction (see SI)
*u*_sdrel,F00x_	Relative standard deviation of the mean of the fluorescence emission spectra of *N* bottles measured with the spectrofluorometer FLS920
*u*_LC,F00x_	Relative uncertainty of the used wavelength correction (see SI)
*u*_r_bb,F00x_	Uncertainty due to inhomogeneities of the bottled RM (see Fig. 3a and b)

Previously, we determined the uncertainty budget of the certified emission spectra of our spectral fluorescence standards by using the sum of the squared uncertainties ${u}_{\text{comb}}=\sqrt{{u}_{1}^{2}+{u}_{2}^{2}+\dots +{u}_{n}^{2}}$, instead of employing partial derivatives. The latter approach is valid when the partial derivatives are equal to 1. This is the case when the measurand equals the sum of the contributing quantities, i.e., $y={x}_{1}+{x}_{2}+\dots +{x}_{n}$. This approach aligns with ISO 33405:2024 [27], where it is used for combining uncertainties from characterization (${u}_{\text{cha}r}$), heterogeneity (${u}_{\text{hom}}$), and if necessary, long- and short-time stability studies, i.e., effects (${u}_{\text{lts}, }{u}_{\text{sts},}$): ${u}_{\text{CRM}}=\sqrt{{u}_{\text{char}}^{2}+{u}_{\text{hom}}^{2}+{u}_{\text{lts}}^{2}{+u}_{\text{sts}}^{2}}$. The sum of the squared uncertainties then approximates the combined uncertainty if the partial derivatives are close to 1. However, when the partial derivatives significantly differ in magnitude, as is the case for the uncertainty budgets obtained for BAM-F007 and BAM-F009, the partial derivatives must be calculated and applied for an accurate uncertainty assessment.

For the calculation of the certified emission spectra, Eqs. 6 and 7 were combined to form Eq. 8.

8 $${I}_{\text{c},\text{F}00\text{x}}= \frac{{I}_{u,F00x}\left(\lambda \right)\cdot {I}_{\text{FRS}1}\left(\lambda \right) \cdot {I}_{\text{SRS}}\left(\lambda \right) \cdot {T}_{\text{Filter}}\left(\lambda \right)\cdot \lambda }{{I}_{\text{U}\_\text{LC}}\left(\lambda \right)}$$

The associated uncertainty translates to Eq. 9.

9 $${u}_{c,F00x}^{2}= {\left(\frac{\partial {I}_{c,F00x}}{\partial {I}_{\text{u},\text{F}00\text{x}}}\right)}^{2}{u}_{\text{u},\text{F}00\text{x}}^{2}+{\left(\frac{\partial {I}_{c,F00x}}{\partial {I}_{\text{FRS}1}}\right)}^{2}{u}_{\text{FRS}1}^{2}+{\left(\frac{\partial {I}_{c,F00x}}{\partial {I}_{\text{SRS}}}\right)}^{2}{u}_{\text{SRS}}^{2}+{\left(\frac{\partial {I}_{c,F00x}}{\partial {T}_{\text{Filter}}}\right)}^{2}{u}_{\text{Filter}}^{2}+{\left(\frac{\partial {I}_{c,F00x}}{\partial {I}_{{\text{U}}_{\text{LC}}}}\right)}^{2}{u}_{\text{U}\_\text{LC}}^{2}$$

Since the certified emission spectra are derived from the measured uncorrected emission spectra of BAM-F007 and BAM-F009, it is necessary to include the uncertainties associated with the measurement of the uncorrected spectra.

10 $${u}_{u,F00x}=\sqrt{{u}_{\text{sdrel},\text{ F}00\text{x}}^{2}\left(\lambda \right)+{u}_{\text{LC},\text{F}00\text{x}}^{2}\left(\lambda \right)}$$

Instrument-related uncertainty contributions of the calibration measurements were summarized to:

11 $${u}_{\text{U}\_\text{LC}}=\sqrt{{u}_{\text{Det}.-\text{Lin}}^{2}+{u}_{\text{sdrel},\text{c}}^{2}+{u}_{\begin{array}{c}LC,c\\ \end{array}}^{2}}$$

**Fig. 5:**
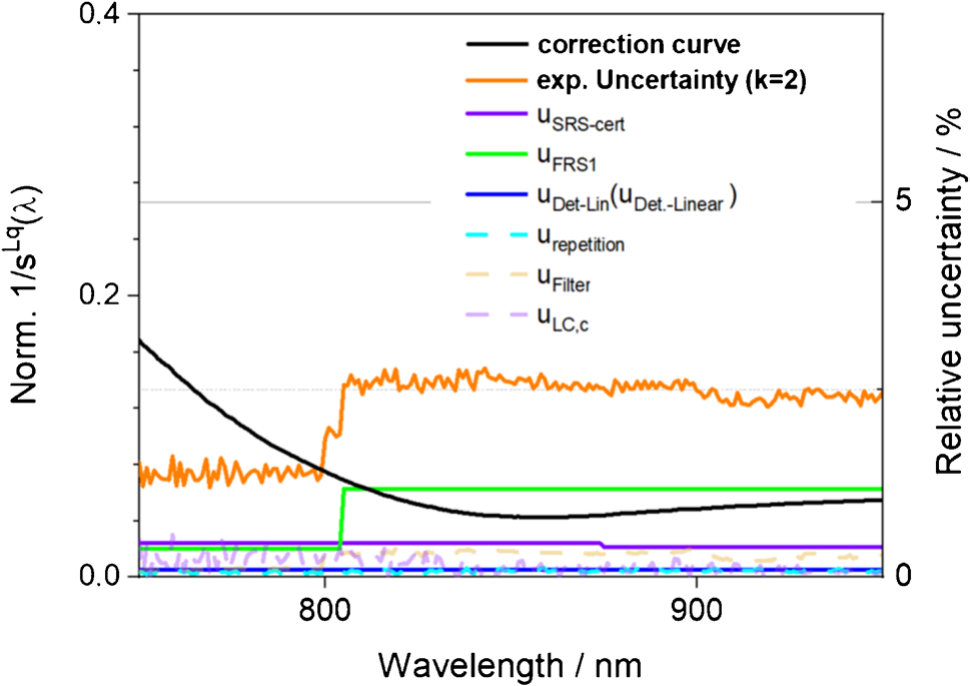
Emission correction curve ${s}^{{L}_{q}}\left({\lambda }_{\text{em}}\right)$ of the calibrated reference fluorescence spectrometer used for the certification including its wavelength-dependent relative uncertainties contributing to the corrected emission spectra of BAM-F007 and BAM-F009

The calibration-related uncertainty contributions are displayed in Fig. 5.

The uncertainty ${u}_{c,F00x}$ corresponds to the characterization uncertainty ${u}_{\text{char}}$. For the combined uncertainty of the corrected emission spectra of BAM-F007 and BAM-F009, the uncertainty originating from possible inhomogeneities of the bottled RM needs to be included into the uncertainty budget according to ISO 33405:2024 [30]. Uncertainties due to a sample instability can be neglected, given the results from the stability studies of both dyes. Since for the homogeneity studies, uncorrected fluorescence spectra were utilized, the homogeneity uncertainty ${u}_{{\text{r}}_{\text{bb}}, F00x}$ had to be transferred to ${I}_{\text{c},\text{F}00\text{x}}$: ${u}_{\text{hom}}^{2}(\lambda )={\left(\frac{\partial {I}_{c,F00x}(\lambda )}{\partial {I}_{{\text{U}}_{\text{LC}}}}\right)}^{2}{u}_{{\text{r}}_{\text{bb}}, F00x}^{2}(\lambda )$. Consequently, the combined uncertainty for the CRM was calculated according to Eq. 11.

12 $${u}_{\text{CRM}}=\sqrt{{u}_{c,F00x}^{2}(\lambda )+{u}_{\text{hom}}^{2}(\lambda )}$$

**Fig. 6:**
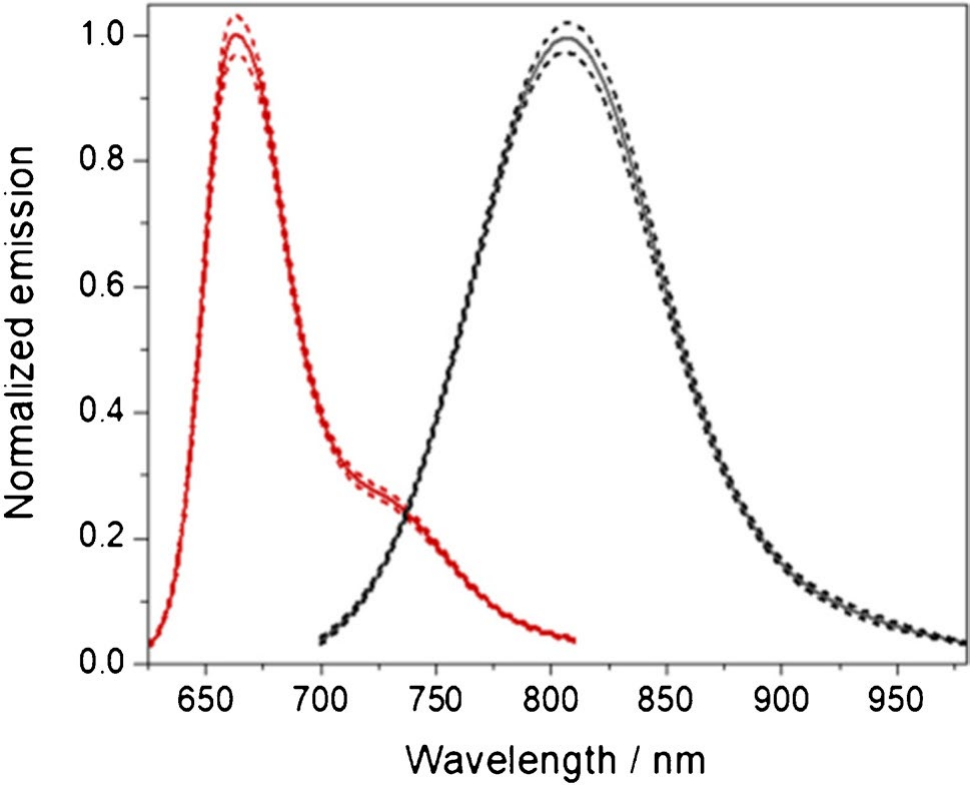
Certified emission spectra of F007 (dark red) and F009 (black) including the corresponding uncertainty bands

The resulting expanded uncertainties (*k* = 2) were then calculated according to Eq. 12. The certified emission spectra of BAM-F007 and BAM-F009 including the respective wavelength-dependent uncertainties are shown in Fig. 6.

13 $${U}_{\text{rel},\text{F}00\text{x}}=2\cdot {u}_{\text{CRM}} / {I}_{\text{c},\text{F}00\text{x}}$$

### Usage of the NIR-extended Calibration Kit spectral fluorescence standards

**Fig. 7:**
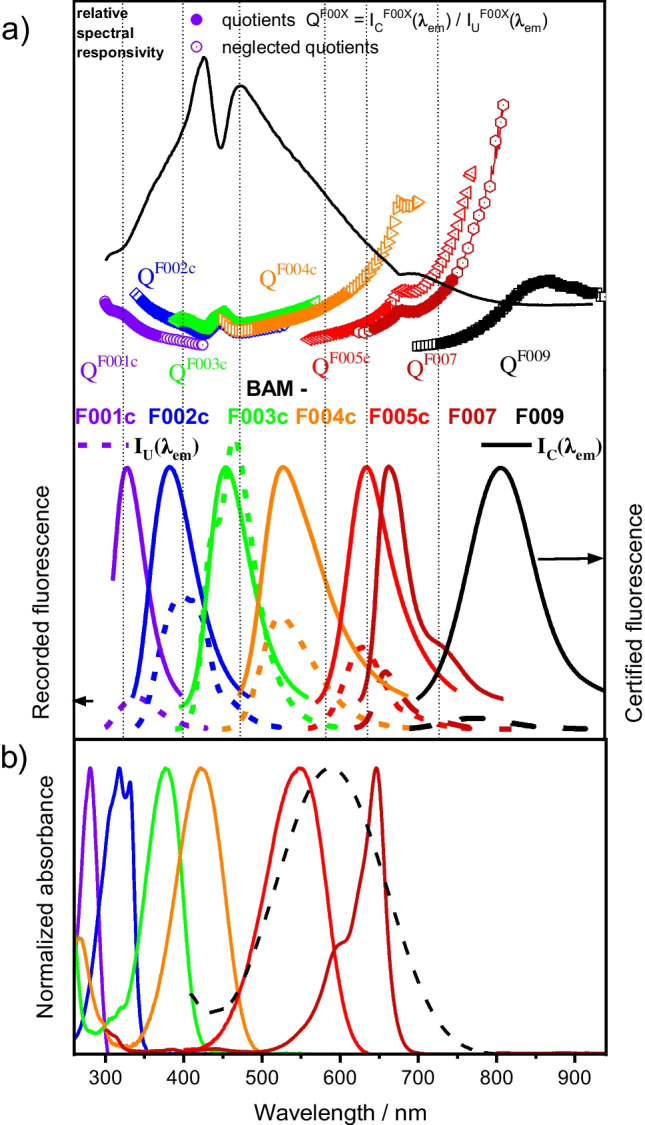
**a** Illustration of the determination of the relative spectral responsivity *s*(*λ*) of a fluorescence instrument with the *Calibration Kit* components BAM-F001b to BAM-F009 and *LinkCorr*. Bottom: certified *normalized corrected fluorescence emission spectra* (solid lines; *I*_C_(*λ*_em_) of BAM-F001b to BAM-F009 and uncorrected, i.e., instrument-dependent emission spectra (dashed lines; *I*_U_(*λ*_em_)), measured with the instrument to be calibrated. Middle: individual quotients Q^F00x^(λ)= *I*_C_(*λ*_em_) / *I*_U_(*λ*_em_), calculated with *LinkCorr* for BAM-F001b to BAM-F009. These quotients equal the inverse relative spectral responsivity 1/*s*(*λ*) of the instrument to be calibrated within the spectral region of the respective spectral fluorescence standard. Top: the finally combined spectral responsivity *s*(*λ*) (solid black line) calculated from the individual quotients. **b** Normalized absorbance and emission spectra of BAM spectral fluorescence standards

The spectral *Calibration Kit* extended now by BAM-F007 and BAM-F009, covering the wavelength region of 580 to 940 nm, enables the determination of the wavelength-dependent relative spectral responsivity *s*(*λ*) of the detection systems of fluorescence instruments in the UV/vis/NIR from about 300 to 940 nm. The simple working principle of this *Calibration Kit* and the software *LinkCorr* developed and validated by BAM is shown in Fig. 7a. The uncorrected (instrument-specific) fluorescence emission spectra of the *Calibration Kit* dyes are thereby determined under conditions used for typical fluorescence measurements. The data evaluation software *LinkCorr* provided by BAM with the spectral *Calibration Kit*, which contains the reference data of the *Calibration Kit* dyes, i.e., their certified normalized corrected emission spectra, then calculates a series of wavelength-dependent quotients Q^F00x^(λ) from the uncorrected fluorescence spectra *I*_U_(*λ*) measured with the fluorescence devices to be calibrated and the certified emission spectra *I*_C_(*λ*) of the *Calibration Kit* dyes and combines these quotients to a global correction curve. This combined correction curve equals the emission correction curve, i.e., the inverse dependent relative spectral responsivity 1/*s*(*λ*) of the emission channel of the fluorescence instrument to be calibrated.

## Conclusion and outlook

In summary, we presented the development, characterization, and certification of two novel near-infrared (NIR) emissive dyes, BAM-F007 and BAM-F009, the certified, spectrally corrected emission of which cover the wavelength range of 580 to 940 nm. Thereby, we derived general design and quality criteria for fluorescence standards used by us for the development of fluorescence standards, and provided possible sources of uncertainty, also with respect to other available fluorescence standards. In addition, procedures for the determination of wavelength-dependent uncertainty budgets of fluorescence emission spectra required for establishing the traceability of fluorescence measurements to the spectral photon radiance scale are provided, including contributions from possible inhomogeneities and instabilities of the bottled spectral fluorescence standards accompanying dye certification at BAM and the calibration of the BAM reference spectrofluorometer characterized with certified physical transfer standards. With this instrument calibration, the traceability of the certified normalized emission spectra to the spectral photon radiance scale is established. These NIR fluorescence standards, which extend the wavelength range of the BAM spectral fluorescence standards BAM-F001b - BAM-F005b from previously 300–750 to now 940 nm and the updated BAM software *LINKCORR*, which can calculate the wavelength-dependent spectral responsivity of the fluorescence instrument to be calibrated from the measured and certified emission spectra of the BAM dyes, will provide the basis for comparable fluorescence measurements in the ultraviolet, visible, and NIR for the fluorescence community in the future. In addition, due to the linkage to the spectral photon radiance scale, which considers the photonic nature of the emitted light, integration of the emission spectra recorded on a fluorescence instrument, calibrated with the BAM spectral fluorescence standards, a calibration with these spectral fluorescence standards can be directly utilized for the relative determination of fluorescence quantum yields. This dye-based spectral calibration procedure is currently expanded to other types of fluorescence measuring devices such as miniaturized fluorescence spectrometers, microplate readers, and fluorescence microscopes.

## Supplementary Information

Below is the link to the electronic supplementary material.

Supplementary file1 (PDF 282 KB)

## Footnotes

1 The terms fluorescence and fluorophore are used for all types of luminescence mechanism and luminophores without explicitly referring to an emission from an excited singlet state.
