# Supplementary material for: Extending certified spectral fluorescence standards for the calibration and performance validation of fluorescence instruments to the NIR—closing the gap from 750 to 940 nm with two novel NIR dyes
Source: Anal Bioanal Chem. 2025 Feb 12;417(12):2563–77. doi: 10.1007/s00216-024-05723-w (PMC12003472; doi:10.1007/s00216-024-05723-w)
Supplement: Supplementary file 1 — Supplementary file1 (PDF 282 KB) [file 216_2024_5723_MOESM1_ESM.pdf]

Supporting Information

Extending Certified Spectral Fluorescence Standards for the Calibration and Performance Validation of Fluorescence Instruments to the NIR – Closing the Gap from 750 nm to 940 nm with Two Novel NIR Dyes

M. Richter,[1] A, Güttler,[1] J. Pauli,[1]K. Vogel,[2] C. Homann, [1] C. Würth,[1,*] and U. Resch-Genger[1,*]

[1]: Division *Biophotonics*, Federal Institut for Materials Research and Testing (BAM), Richard-Willstaetter-Str. 11, D-12489 Berlin, Germany; christian.wuerth@bam.de; ute.resch@bam.de
[2]: Division *eScience*, Federal Institute for Materials Research and Testing (BAM), Unter den Eichen 87, D-12205 Berlin, Germany

Wavelength correction

The wavelength accuracy of the emission detection monochromator of the fluorescence spectrometer needs to be determined prior to a calibration (for determination of the relative spectral responsivity $s(\lambda_{\text{em}})$) and prior to the certification measurements. If the wavelength accuracy of the emission detection monochromator is adjusted, this takes place prior to the calibration only, to not interfere with the obtained results of the calibration.
Therefore, a low-pressure discharge lamp filled with a gas mixture of mercury (Hg) and argon (Ar) is used (Model: HG-2 Mercury-Argon [UV-NIR], Ocean Optics Inc.), providing i distinct emission lines covering the spectral region from 253 – 1700 nm. The exact spectral position of the emission lines is given by the NIST – Standard Reference Database (National Institute of Standards and Technology, USA). The determined deviations from the listed values are considered for the calibration, as well as for the certification and are calculated using Equation S1.

$$\Delta\lambda_{\text{Peak},i} = \lambda_{\text{Peak},i,\text{meas}} - \lambda_{\text{Peak},i,\text{ref}} \qquad \text{Eq. S1}$$

Prior to the calibration the wavelength accuracy at “time of calibration” was determined as follows.
The emission spectrum of the HG-2-Lamp was measured at the sample position with a 0.4 nm emission monochromator slit width and a 0.2 nm step size for high resolution in the spectral range of 250 – 1100 nm. The deviations $\Delta\lambda_{\text{Peak},i}$ of the positions of the measured lines $\lambda_{\text{Peak},i,\text{meas}}$ to the known listed distinct lines $\lambda_{\text{Peak},i,\text{ref}}$ for Hg and Ar are exemplarily shown in Figure S1.
Prior to the certification measurements the wavelength accuracy at “time of certification” was determined using identical parameters and instrument settings.
Each parabolic distribution of the determined deviations $\Delta\lambda_{\text{Peak},i}$ was fitted using a polynomial regression ($\Delta\lambda_{\text{PolyFit}}$, see Figure S1).

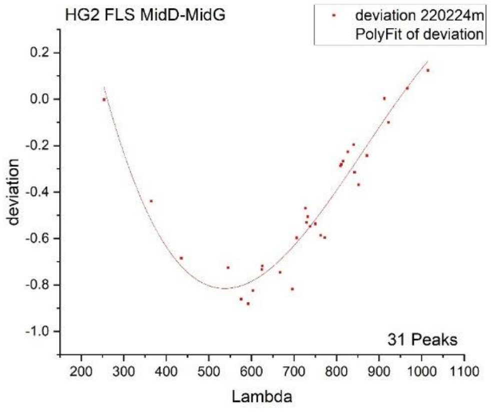

Fig. S1: Determined wavelength deviations of the UV-NIR emission monochromator (“MidG”) of the reference spectrofluorometer FLS920 at “time of calibration” and the fitted regression curve.

The resulting regression curves are used for the correction of the measured emission spectrum of the *Spectral Radiance Standard* – "SRS" $I_U(\lambda_{em})$ and the measured fluorescence spectra $I_{u,F00x}(\lambda)$ for the certification according to Chapter 6.2.1 applied as a correction function on the wavelength scale using Equation S2.

$$\lambda_{corr} = \lambda_{meas} - \Delta\lambda_{PolyFit} \qquad \text{Eq. S2}$$

The determination of the deviations and the use of their polynomial regression contributes to the uncertainty as u_{LC}, which is determined as follows.
The uncertainty of the wavelength correction u_{LC} depends on the resolution used during the measurements (0.2 nm step size), the accuracy of the determination of the peak position, the quality of the polynomial regressing fit and the error resulting from the regression curve. The fit quality can be determined by the analysis of the corresponding residuals (see example Figure S3).

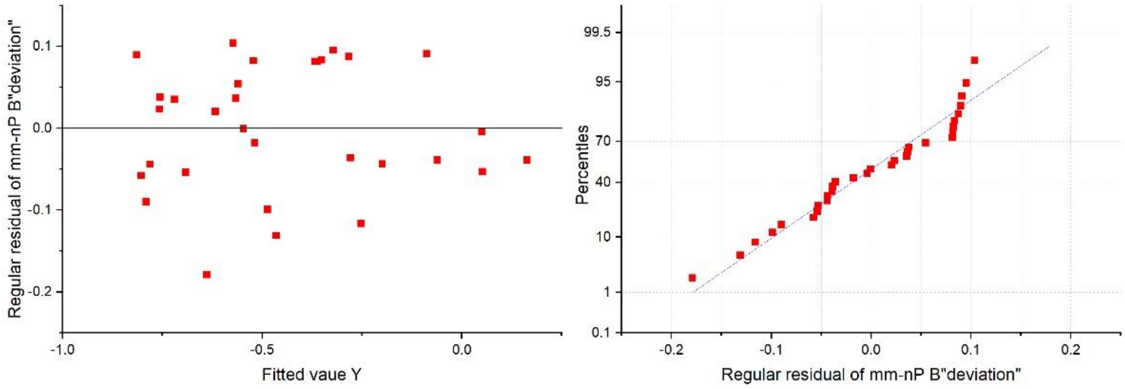

Fig. S2: Residual plots of the polynomial regression fit of the wavelength deviations "time of calibration" as shown in Figure S1.

The residuals are distributed mostly between +/- 0.1 nm deviation from the determined values and can therefore be used for the determination of the uncertainty u_{LC}.
To determine u_{LC} the regression curve is shifted in both directions of the residual distribution, +0.1 nm, and -0.1 nm. The original regression curve and both shifted regression curves are then used for the wavelength correction of the measured spectra. The three resulting spectra are used to determine u_{LC}. Equation S3 exemplarily shows the calculation of $u_{LC,c}$, the uncertainty contribution of the wavelength correction on the correction of the measured emission spectrum of the "SRS" $I_U(\lambda_{em})$

$$u_{LC,c} = \frac{|I_{U\_LC+0.1}(\lambda_{em}) - I_{U\_LC}(\lambda_{em})| + |I_{U\_LC-0.1}(\lambda_{em}) - I_{U\_LC}(\lambda_{em})|}{I_{U\_LC}(\lambda_{em})} \qquad \text{Eq. S3}$$

$I_{U\_LC}(\lambda_{em})$ measured uncorrected emission spectrum of the "SRS" corrected with $PolyFit_{Offset}$

$I_{U\_LC+0.1}(\lambda_{em})$ measured uncorrected emission spectrum of the "SRS" corrected with $PolyFit_{Offset} + 0.1$

$I_{U\_LC-0.1}(\lambda_{em})$ measured uncorrected emission spectrum of the "SRS" corrected with $PolyFit_{Offset} - 0.1$
